# Clinical Outcomes of the Germline *RET* M918T Pathogenic Variant in Hereditary Medullary Thyroid Carcinoma: A Systematic Review and Meta-Analysis

**DOI:** 10.3390/medicina62091636

**Published:** 2026-08-26

**Authors:** Jurairat Jongthawin, Wanlaya Naowaratwattana, Jongkonnee Thanasai, Darunee Puangpronpitag, Hasaya Dokduang, Issarapong Phosuk, Wipavadee Daiponmak

**Affiliations:** 1 Faculty of Medicine, Mahasarakham University, Maha Sarakham 44000, Thailand; jurairat.j@msu.ac.th (J.J.); wanlaya.n@msu.ac.th (W.N.); jongkonnee@msu.ac.th (J.T.); darunee.p@msu.ac.th (D.P.); hasaya.d@msu.ac.th (H.D.); 2 Biomedical Science Research Unit, Mahasarakham University, Maha Sarakham 44000, Thailand; 3 International and National Collaborative Network and Innovation for Community Health Development Research Unit, Mahasarakham University, Maha Sarakham 44000, Thailand; 4 Department of Public Health, Amnatcharoen Campus, Mahidol University, Amnat Charoen 37000, Thailand; issarapong.pho@mahidol.ac.th

**Keywords:** germline *RET* M918T pathogenic variant, hereditary medullary thyroid carcinoma, lymph node metastasis, metastatic disease, prognosis, systematic review, meta-analysis

## Abstract

*Background and Objectives*: Hereditary medullary thyroid carcinoma (hMTC) exhibits substantial clinical heterogeneity according to the underlying germline *RET* pathogenic variant. Although the germline *RET* M918T pathogenic variant is associated with aggressive disease, its associations with clinical outcomes have not been comprehensively quantified. We aimed to evaluate the associations between the M918T variant and adverse clinical outcomes in patients with hMTC. *Materials and Methods*: PubMed, Scopus, and Embase were searched from database inception to May 2026 in accordance with PRISMA 2020. Eligible studies compared patients with the germline *RET* M918T pathogenic variant with those harboring other germline *RET* pathogenic variants. Random-effects models were used to pool odds ratios (ORs) and 95% confidence intervals (CIs) for lymph node metastasis, metastatic disease, advanced-stage disease, and mortality. Risk of bias was assessed using the Quality in Prognosis Studies tool. The protocol was prospectively registered in PROSPERO (CRD420261377120). *Results*: Nine cohort studies involving 1485 participants were included, with outcome-specific sample sizes varying. Three studies had a low overall risk of bias and six had a moderate risk. The M918T variant was associated with increased odds of lymph node metastasis (OR 7.86, 95% CI 2.58–23.94), metastatic disease (OR 6.50, 95% CI 3.72–11.37), advanced-stage disease (OR 4.88, 95% CI 2.30–10.33), and mortality (OR 7.32, 95% CI 3.14–17.10). The association was stronger for disease-specific than all-cause mortality, although the subgroup difference was not statistically significant. *Conclusions*: The germline *RET* M918T pathogenic variant was associated with adverse clinical outcomes compared with other germline *RET* pathogenic variants. However, heterogeneous comparator groups and residual confounding preclude causal interpretation.

## 1. Introduction

Medullary thyroid carcinoma (MTC) is a rare neuroendocrine malignancy arising from the parafollicular C cells of the thyroid gland and accounts for approximately 1–2% of all thyroid cancers [1,2]. Despite its relatively low incidence, MTC contributes disproportionately to thyroid cancer-related mortality because of its propensity for regional lymph node involvement, distant metastasis, persistent disease, and recurrence [1,2]. Approximately 20–25% of MTC cases occur in the hereditary setting and are caused by activating germline *RET* pathogenic variants [1,2,3]. Hereditary MTC (hMTC) occurs within the multiple endocrine neoplasia type 2 (MEN2) spectrum, which comprises MEN2A and MEN2B (also termed MEN3); familial MTC is considered a phenotypic variant of MEN2A [1,3].

The *RET* proto-oncogene encodes a transmembrane receptor tyrosine kinase involved in cellular proliferation, differentiation, migration, and survival [2,4]. Activating germline RET pathogenic variants are the principal molecular drivers of hMTC and are associated with substantial genotype–phenotype heterogeneity, influencing disease penetrance, age at onset, metastatic potential, and clinical outcomes [1,2,3,4]. The germline *RET* c.2753T > C (p.Met918Thr; M918T) pathogenic variant, described using Human Genome Variation Society nomenclature [5] and located at codon 918 in exon 16, is the predominant genetic alteration associated with MEN3 and is classified in the American Thyroid Association highest-risk category (ATA-HST) [1,2,3]. M918T is associated with MTC arising during infancy or early childhood and with an increased risk of early lymph node and distant metastases [1,6,7].

Current management of hMTC is informed by germline *RET* genotype. The revised ATA guidelines recommend genetic testing soon after birth in infants at risk of MEN3 and thyroidectomy during the first year of life, potentially within the first months, for children carrying M918T [1]. The European Society for Medical Oncology similarly recommends total thyroidectomy within the first year of life for germline M918T carriers, whereas the European Thyroid Association emphasizes genetic counseling, germline *RET* testing, and cascade testing of at-risk relatives to facilitate early genotype-informed management [8,9]. DNA-based family screening can identify germline *RET* carriers before conventional clinical or biochemical screening and thereby enable earlier preventive management [8,10]. The ATA further recommends that children with MEN2A or MEN3 be managed by experienced physicians and surgeons in tertiary-care centers [1,11]. In infants without suspicious lymph nodes, central neck dissection should be individualized according to the feasibility of identifying and preserving or autotransplanting the parathyroid glands. Pheochromocytoma should also be excluded before thyroid surgery or another interventional procedure in patients with MEN2 [1]. These recommendations are based predominantly on observational evidence and expert consensus because germline genotype is an inherent, non-assignable exposure.

Although M918T is already recognized as the highest-risk germline *RET* pathogenic variant, the magnitude of its association with specific adverse clinical outcomes relative to other germline *RET* pathogenic variants has not been comprehensively quantified. Comparative cohort studies have reported associations between M918T and lymph node metastasis, metastatic disease, advanced-stage disease, recurrence, and mortality; however, the magnitude and consistency of these associations remain uncertain because of differences in study design, sample size, age distribution, case ascertainment, surgical timing, outcome definitions, and comparator variant composition [12,13,14,15,16,17,18,19,20].

To the best of our knowledge, no previous systematic review and meta-analysis has specifically synthesized the associations between the germline *RET* M918T pathogenic variant and MTC-related clinical outcomes in hMTC. Given the rarity of hMTC and the small sample sizes of individual cohorts, quantitative synthesis may provide more precise estimates of these associations. Therefore, this study aimed to evaluate the associations of M918T with lymph node metastasis, metastatic disease, advanced-stage disease, and mortality by comparing M918T carriers with study-specific comparator groups carrying other germline *RET* pathogenic variants. This synthesis was intended to characterize the prognostic implications of M918T within its established ATA-HST classification, rather than to establish new recommendations for surgery, surveillance, or treatment.

## 2. Materials and Methods

### 2.1. Protocol and Registration

The protocol for this systematic review and meta-analysis was prospectively registered with the International Prospective Register of Systematic Reviews (PROSPERO; registration number CRD420261377120). No amendments to the registered protocol were made after registration. This review was conducted and reported in accordance with the Preferred Reporting Items for Systematic Reviews and Meta-Analyses (PRISMA) guidelines [21].

### 2.2. Search Strategy

A comprehensive literature search was conducted in PubMed, Embase, and Scopus from database inception to 30 May 2026. The search strategy combined controlled vocabulary terms, including Medical Subject Headings (MeSH) in PubMed and Emtree terms in Embase, with free-text terms related to medullary thyroid carcinoma, *RET* variants, and clinical or prognostic outcomes using Boolean operators. The core search concept was (“medullary thyroid carcinoma” OR “medullary thyroid cancer” OR “MTC”) AND (“*RET*” OR “*RET* mutation” OR “*RET* mutations” OR “*RET* proto-oncogene” OR “germline *RET*” OR “somatic *RET*”) AND (“prognosis” OR “survival” OR “disease-free survival” OR “recurrence” OR “metastasis” OR “mortality”). No language or publication-year filters were applied at the database-search stage. During study selection, eligibility was restricted to human studies published in English from January 2000 onward that reported relevant clinical outcomes among carriers of germline *RET* pathogenic variants. Studies reporting M918T solely as a somatic tumor variant were excluded. The reference lists of included studies and relevant review articles were manually screened, but no additional records were identified through this process. The complete database-specific search strategies are presented in Supplementary Table S1.

### 2.3. Eligibility Criteria

Study eligibility was determined using predefined inclusion and exclusion criteria based on the PECO framework [22]. The population (P) comprised patients with hMTC or germline *RET* carriers with evaluable MTC-related outcomes. The exposure (E) was the germline *RET* M918T pathogenic variant, whereas the comparator (C) comprised study-specific groups carrying other germline *RET* pathogenic variants. Comparator groups could include individual variants, codon-based groups, or genotype-based risk categories, as defined in the original studies. The outcomes (O) were lymph node metastasis, metastatic disease, advanced-stage disease, and mortality. Other endocrine and non-endocrine manifestations associated with MEN3 were outside the predefined scope of this review.

Eligible studies were comparative observational studies, including cohort studies and comparative case series, that reported at least one predefined outcome separately for the exposure and comparator groups and provided sufficient group-specific data to calculate odds ratios (ORs) and corresponding 95% confidence intervals (CIs). Only full-text articles published in English were eligible. No restrictions were imposed on participant age or study sample size because hMTC and cohorts of germline M918T carriers are rare.

Reviews, editorials, conference abstracts, case reports, non-English full-text articles, animal and in vitro studies, studies restricted to sporadic MTC, noncomparative studies, and studies with insufficient group-specific outcome data were excluded.

### 2.4. Exposure Definition

The exposure of interest was the germline *RET* c.2753T>C (p.Met918Thr; M918T) pathogenic variant at codon 918 in exon 16. Germline status and variant identification were based on the molecular genetic testing methods reported in the original studies. No restriction was imposed on the testing platform, provided that germline M918T and comparator variants could be reliably distinguished and group-specific outcome data were extractable. Testing of peripheral blood or another constitutional DNA source was accepted as evidence of germline status. When the testing method or specimen source was not reported, a study remained eligible only if the authors explicitly identified the *RET* variants as germline or clearly documented the hereditary nature of the study population. Studies reporting M918T solely as a somatic tumor variant were excluded.

Study-specific comparator variants and group definitions were extracted and harmonized, and variant descriptions were standardized according to Human Genome Variation Society nomenclature when sufficient information was available [5]. Comparator variants were eligible if they were reported as disease-causing or pathogenic germline *RET* variants in the original studies or were recognized as MEN2-associated variants in the revised American Thyroid Association guidelines for the management of medullary thyroid carcinoma [1]. Variants reported solely as variants of uncertain significance were not eligible. Because this review did not perform variant-level reassessment, individual variants were not independently reclassified according to the American College of Medical Genetics and Genomics/Association for Molecular Pathology criteria [23].

### 2.5. Study Selection and Data Extraction

All retrieved records were imported into EndNote version 21.0 (Clarivate Analytics, Philadelphia, PA, USA), and bibliographic duplicates were removed. Two reviewers independently screened titles and abstracts and subsequently assessed full-text reports against the predefined eligibility criteria. Reasons for exclusion at the full-text stage were recorded. Two reviewers independently extracted data into Microsoft Excel 2021 (Microsoft Corporation, Redmond, WA, USA) using a standardized, pilot-tested form. Extracted variables included study and population characteristics; age category; ages at *RET* variant identification, MTC diagnosis, and thyroidectomy; follow-up duration; genetic testing method and specimen source; exposure and comparator group sizes; comparator variants or risk categories; indications for thyroidectomy; case ascertainment methods; and outcome data and definitions. Variables not explicitly reported were recorded as “not reported” and were not inferred from related variables. Characteristics reported only for the overall cohort were recorded at the study level and were not used in variant-specific subgroup analyses.

Event counts and corresponding group-specific denominators were extracted separately for lymph node metastasis, metastatic disease, advanced-stage disease, and mortality. Lymph node metastasis was defined as regional lymph node involvement. Metastatic disease was defined as M1 disease at diagnosis, distant metastasis during follow-up, or distant recurrence. Advanced-stage disease was defined as American Joint Committee on Cancer stage III–IV, as reported in the original studies. Mortality was classified as disease-specific or all-cause according to the definition used in each original study. Reported outcome definitions were mapped to the prespecified review outcome categories before quantitative synthesis.

Potential overlap among study populations was assessed using study setting, recruitment period, authorship, participant characteristics, *RET* variant distribution, and reported outcomes. For each outcome-specific pooled analysis, the dataset with the most complete relevant outcome data was retained; when outcome completeness was comparable, the dataset with the largest relevant sample was selected. Disagreements regarding study selection, data extraction, outcome classification, or potentially overlapping populations were resolved by consensus or consultation with a third reviewer.

### 2.6. Risk of Bias Assessment

The risk of bias of the included studies was independently assessed by two reviewers using the Quality in Prognosis Studies (QUIPS) tool, which is specifically designed for systematic reviews of prognostic factor studies [24]. The QUIPS tool evaluates six methodological domains: (i) study participation, (ii) study attrition, (iii) prognostic factor measurement, (iv) outcome measurement, (v) study confounding, and (vi) statistical analysis and reporting. Each domain was rated as having a low, moderate, or high risk of bias according to the QUIPS guidance. An overall risk-of-bias judgment was assigned to each study based on the ratings across all six domains. Studies were classified as having a low overall risk of bias when most domains were rated as low risk, a moderate overall risk of bias when one or more domains had methodological limitations that were unlikely to substantially affect the validity of the results, and a high overall risk of bias when one or more domains were judged to have a high risk of bias likely to compromise the validity of the findings. Disagreements between reviewers were resolved through discussion and consensus or, when necessary, by consultation with a third reviewer. The results of the risk-of-bias assessment were summarized descriptively and considered during the interpretation of the pooled effect estimates.

### 2.7. Statistical Analysis

Meta-analyses evaluated the associations between the germline *RET* M918T pathogenic variant and clinical outcomes in patients with hMTC. Unadjusted odds ratios (ORs) and 95% confidence intervals (CIs) were calculated from event counts and group-specific sample sizes for M918T carriers and study-specific comparator groups carrying other germline *RET* pathogenic variants. Because the comparator groups varied across studies and could comprise individual variants, codon-based groups, or genotype-based risk categories, the reference category did not represent a single uniform variant group. Separate analyses were conducted for lymph node metastasis, metastatic disease, advanced-stage disease, and mortality, including only studies with sufficiently comparable outcome definitions. Pooled estimates were calculated using random-effects models to account for anticipated clinical and methodological heterogeneity, with common-effect estimates presented for comparison [25,26]. Heterogeneity was assessed using Cochran’s Q test and the I^2^ statistic and interpreted as low (<25%), moderate (25% to <50%), substantial (50% to <75%), or considerable (≥75%) [27]. To avoid duplicate patient inclusion, only the dataset providing the most complete outcome-specific data was retained from potentially overlapping cohorts. Leave-one-out sensitivity analyses were performed to assess the influence of individual studies. Mortality was analyzed according to outcome definition. Subgroup analyses by thyroidectomy indication and case ascertainment were not performed because these characteristics were not consistently reported separately by variant group and outcome; other subgroup analyses were not undertaken because of the limited number of studies. Publication bias was not formally assessed because fewer than 10 studies contributed to each outcome. Analyses were performed in R using the meta package, with RStudio version 2024.04.2+764 (Posit Software, PBC, Boston, MA, USA) as the integrated development environment [28].

## 3. Results

### 3.1. Search Results

**Figure 1:**
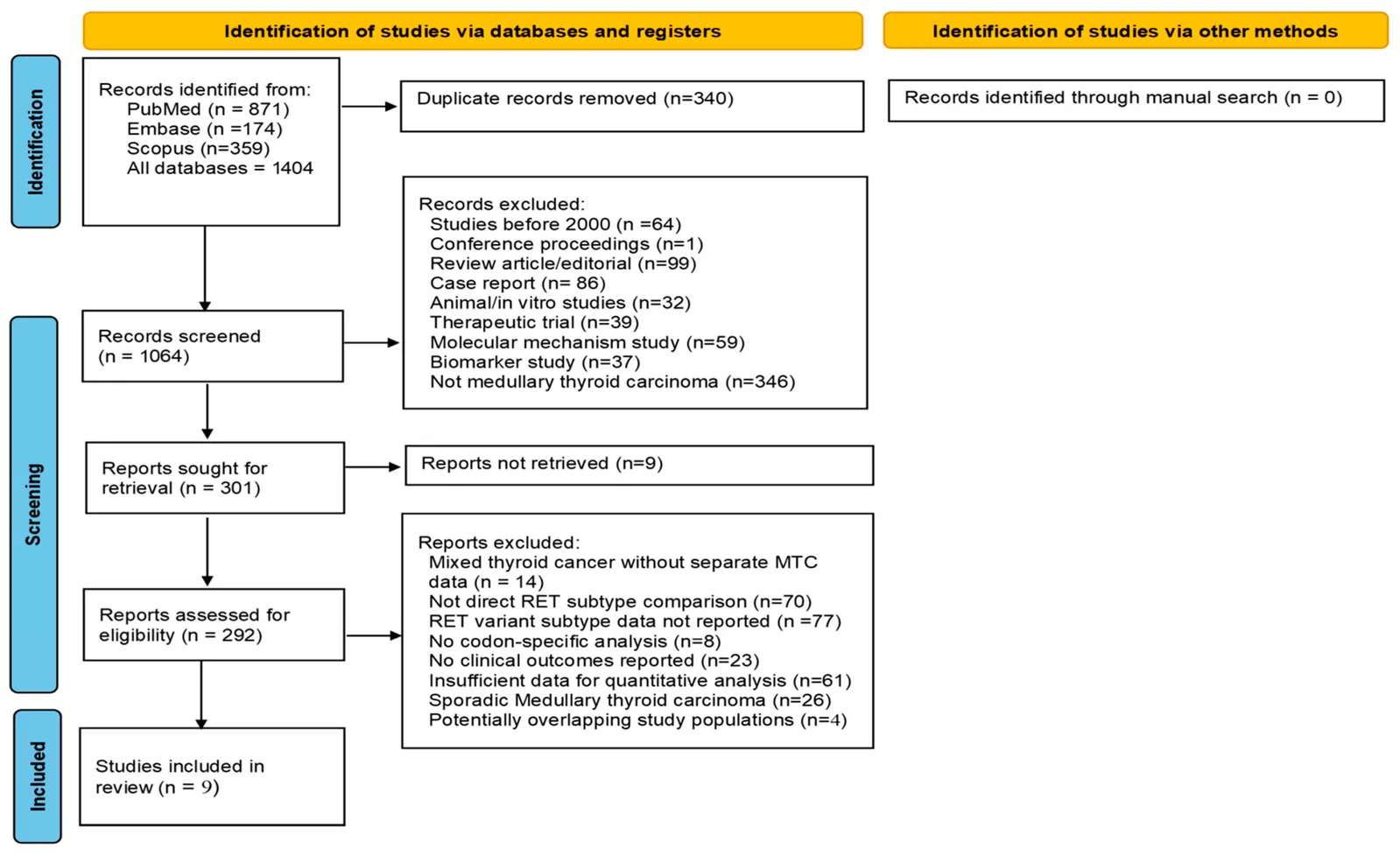
The PRISMA 2020 flow diagram outlines the process of study selection for the review.

The study selection process is presented in Figure 1. The database searches identified 1404 records: 871 from PubMed, 174 from Embase, and 359 from Scopus. Manual screening of the reference lists of included studies and relevant review articles identified no additional records (*n* = 0). After 340 duplicate records were removed, 1064 records underwent title and abstract screening, of which 763 were excluded. The full texts of 301 reports were sought for retrieval; a total of 9 reports could not be retrieved because their full texts were unavailable, leaving 292 reports for eligibility assessment. Of these, 283 were excluded: mixed thyroid cancer populations without separate MTC data (*n* = 14), no direct comparison between *RET* variant subtypes (*n* = 70), no *RET* variant subtype data (*n* = 77), no codon-specific analysis (*n* = 8), no relevant clinical outcomes (*n* = 23), insufficient data for meta-analysis (*n* = 61), restriction to sporadic MTC (*n* = 26), and potentially overlapping study populations (*n* = 4). Potential overlap was assessed based on similarities in study setting, recruitment period, authorship, participant characteristics, sample size, *RET* variant distribution, and reported outcomes. Because patient-level identifiers were unavailable, overlap could not be definitively confirmed. For each potentially overlapping population, the report providing the most complete data for the predefined outcomes was retained to minimize the risk of double-counting. Ultimately, nine studies met the eligibility criteria and were included in the systematic review and meta-analysis.

### 3.2. Characteristics of the Included Studies

The characteristics of the included studies are summarized in Table 1. Nine cohort studies published between 2003 and 2026 were included: eight retrospective cohorts and one prospective multicenter registry cohort. The studies were conducted in the United States, France, Germany, Italy, India, and South Korea, with cohort sizes ranging from 23 to 708 participants. The study populations included pediatric, adolescent, and adult patients with hMTC carrying germline *RET* pathogenic variants.

Age at MTC diagnosis and age at thyroidectomy were reported in three and six studies, respectively; none reported age at germline *RET* variant identification. Four studies described the genetic testing method, including DNA sequencing, allele-specific hybridization, PCR, and direct DNA sequencing, with no explicit use of NGS. Peripheral blood was identified as the specimen source in four studies, whereas the testing method and/or specimen source was not reported in the remaining studies.

All studies provided variant-specific data permitting direct comparisons between germline *RET* M918T and other germline *RET* pathogenic variants. However, comparator groups varied across studies and included codon 634 variants, ATA high- or moderate-risk variants, or broader groups of other germline *RET* variants. Thyroidectomy indication and case ascertainment were inconsistently reported and were not generally available separately by variant group; therefore, stratified analyses by prophylactic versus therapeutic thyroidectomy or index versus family-screened status were not feasible. TNM or pathological TNM information was reported in six studies, although staging was variably assessed at diagnosis, presentation, or postoperative pathological examination. Genotype-specific 5-year survival estimates were reported in only two studies and were therefore insufficient for separate quantitative synthesis. Study-specific other germline *RET* variant comparators, thyroidectomy indications, and case ascertainment are provided in Supplementary Table S2. Reported outcomes included lymph node metastasis, metastatic disease, advanced-stage disease, and mortality.

### 3.3. Quality of the Included Studies

The risk-of-bias assessment is summarized in Supplementary Table S3. Using the Quality in Prognosis Studies (QUIPS) tool, three studies had a low overall risk of bias and six had a moderate overall risk; none had a high risk. The Prognostic Factor Measurement and Outcome Measurement domains were generally rated as low risk because germline *RET* variant status and clinical outcomes were assessed using established genetic and clinical criteria. The main concerns involved Study Attrition, Study Confounding, and Statistical Analysis and Reporting, primarily because of incomplete adjustment for potential confounders and limitations inherent in retrospective cohort designs.

### 3.4. Association Between the M918T Variant and Lymph Node Metastasis

**Figure 2:**
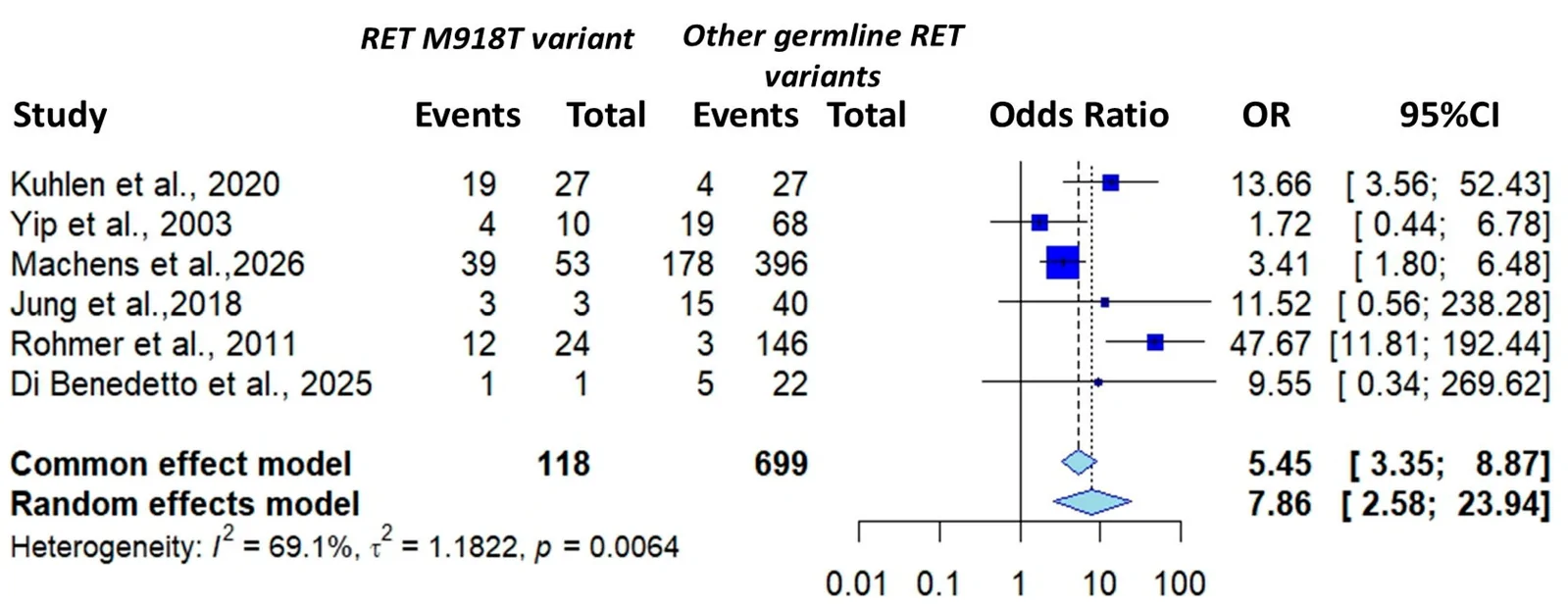
Forest plot of pooled odds ratios (ORs) for lymph node metastasis comparing the M918T variant with other germline *RET* variants in hMTC. Squares represent study-specific estimates, horizontal lines indicate 95% confidence intervals (CIs), and diamonds represent pooled estimates from the common-effect and random-effects models [12,13,14,17,19,20].

The pooled analysis of lymph node metastasis is presented in Figure 2. The M918T variant was associated with higher odds of lymph node metastasis than other germline *RET* variants (OR 7.86, 95% CI 2.58–23.94). Between-study heterogeneity was substantial (I^2^ = 69.1%; *p* = 0.0064), indicating variation in effect magnitude across studies. Nevertheless, most studies showed an effect direction consistent with increased odds of lymph node involvement in the M918T group.

### 3.5. Association Between the M918T Variant and Metastatic Disease

**Figure 3:**
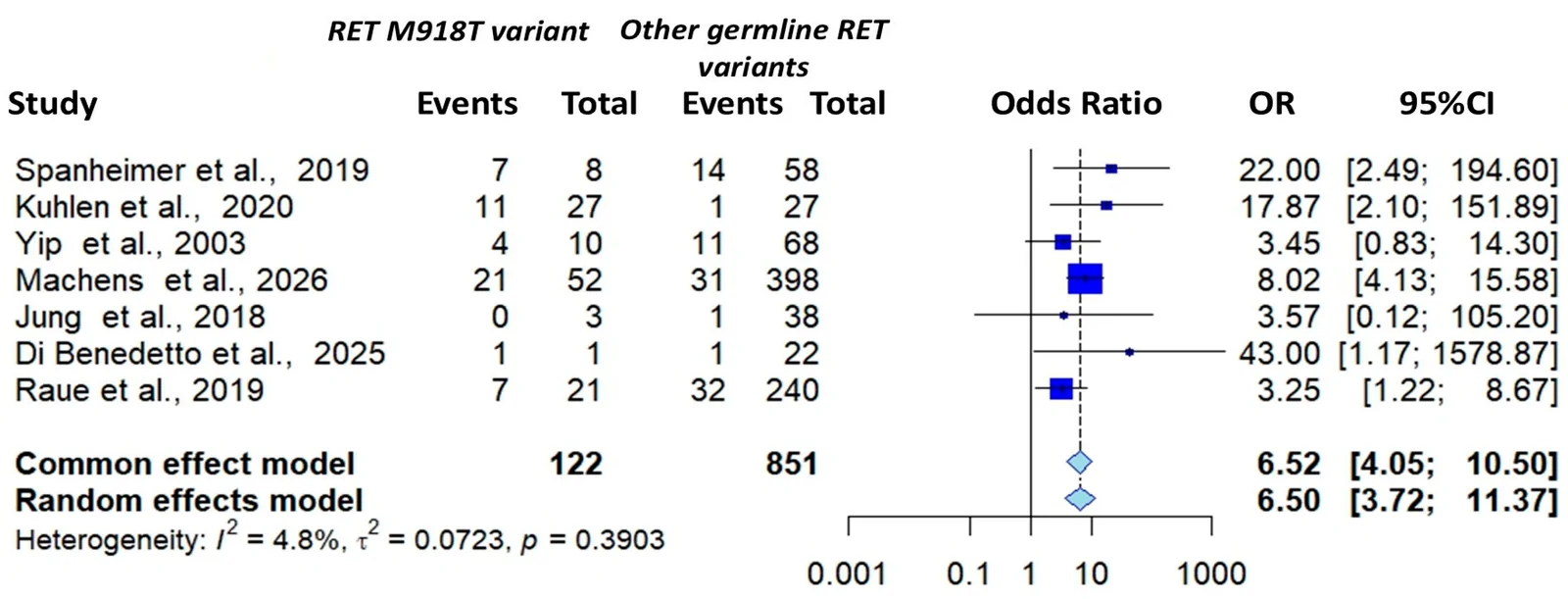
Forest plot of pooled odds ratios (ORs) for metastatic disease comparing the M918T variant with other germline *RET* variants in hMTC. Squares represent study-specific estimates, horizontal lines indicate 95% confidence intervals (CIs), and diamonds represent pooled estimates from the common-effect and random-effects models [12,14,15,16,17,19,20].

The pooled analysis of metastatic disease is presented in Figure 3. The M918T variant was associated with higher odds of metastatic disease than other germline *RET* variants (OR 6.50, 95% CI 3.72–11.37). Between-study heterogeneity was low (I^2^ = 4.8%; *p* = 0.3903), indicating consistent findings across studies. Despite differences in study size and event frequency, the direction and magnitude of the associations were generally consistent.

### 3.6. Association Between the M918T Variant and Advanced-Stage Disease

**Figure 4:**
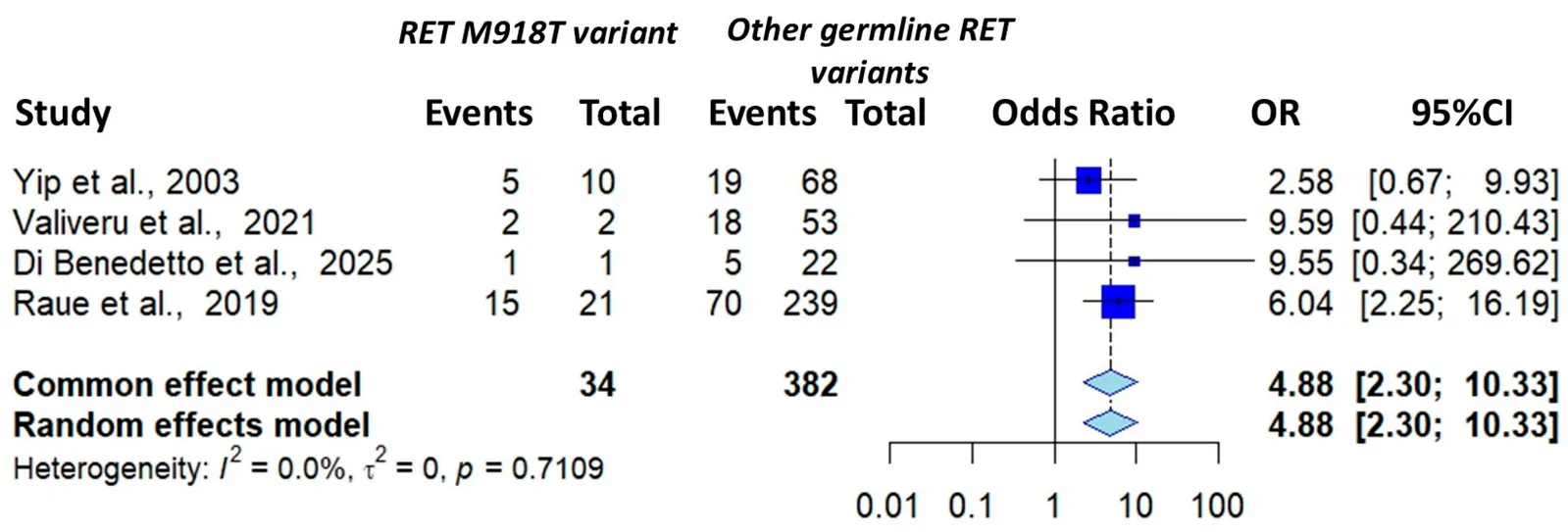
Forest plot of pooled odds ratios (ORs) for advanced-stage disease comparing the M918T variant with other germline *RET* variants in hMTC. Squares represent study-specific estimates, horizontal lines indicate 95% confidence intervals (CIs), and diamonds represent pooled estimates from the common-effect and random-effects models [12,16,18,19].

The pooled analysis of advanced-stage disease is presented in Figure 4. The M918T variant was associated with higher odds of advanced-stage disease than other germline *RET* variants (OR 4.88, 95% CI 2.30–10.33). No between-study heterogeneity was observed (I^2^ = 0.0%; *p* = 0.7109), indicating consistent findings across studies. Although the study-specific estimates varied in magnitude, their direction consistently indicated higher odds of advanced-stage disease in the M918T group.

### 3.7. Association Between the M918T Variant and Mortality

**Figure 5:**
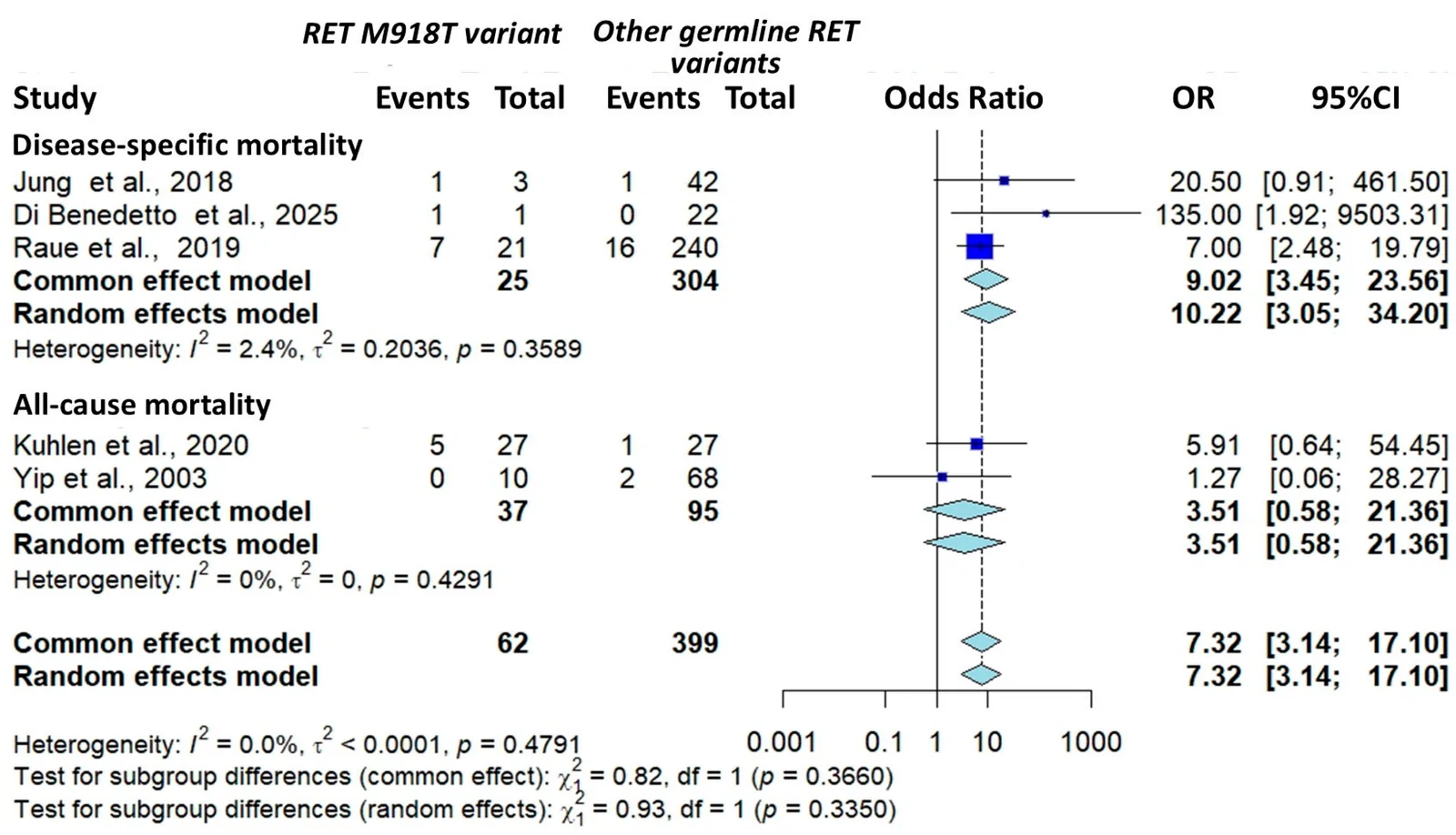
Forest plot of the subgroup meta-analysis of mortality comparing the M918T variant with other germline *RET* variants in hMTC. Mortality was stratified as disease-specific or all-cause mortality. Squares represent study-specific estimates, horizontal lines indicate 95% confidence intervals (CIs), and diamonds represent pooled estimates from the common-effect and random-effects models [12,14,16,17,19].

The pooled analysis of mortality is presented in Figure 5. The M918T variant was associated with higher odds of mortality than other germline *RET* variants (OR 7.32, 95% CI 3.14–17.10), with no between-study heterogeneity (I^2^ = 0.0%; *p* = 0.4791). In subgroup analyses, the M918T variant was associated with higher odds of disease-specific mortality (OR 10.22, 95% CI 3.05–34.20; I^2^ = 2.4%), whereas the association with all-cause mortality was not statistically significant (OR 3.51, 95% CI 0.58–21.36; I^2^ = 0.0%). The difference between mortality subgroups was not statistically significant (*p* = 0.3350).

### 3.8. Sensitivity Analysis

Leave-one-out sensitivity analyses were performed to assess the robustness of all pooled estimates. For lymph node metastasis, the sequential exclusion of individual studies did not materially alter the results, with pooled ORs ranging from 4.65 to 11.14; all estimates remained statistically significant (all *p* < 0.01). The overall association remained significant (OR = 7.86, 95% CI 2.58–23.94; *p* = 0.0003), although heterogeneity varied across iterations (I^2^ = 28.8–75.1%). Similarly, for metastatic disease, pooled ORs ranged from 5.93 to 8.09 and remained statistically significant in all iterations (all *p* < 0.0001). The pooled estimate remained stable (OR = 6.50, 95% CI 3.72–11.37; *p* < 0.0001), with consistently low heterogeneity (I^2^ = 0–19.1%). For advanced-stage disease, pooled ORs ranged from 3.63 to 6.50, and all analyses remained significant (all *p* ≤ 0.0291). The overall association remained robust (OR = 4.88, 95% CI 2.30–10.33; *p* < 0.0001), with no observed heterogeneity (I^2^ = 0%). For mortality outcomes, sequential omission of individual studies yielded pooled ORs ranging from 6.49 to 8.44, with all analyses remaining statistically significant (all *p* ≤ 0.0054). The overall pooled estimate remained significant (OR = 7.32, 95% CI 3.14–17.10; *p* < 0.0001), with no evidence of between-study heterogeneity (I^2^ = 0.0%). Overall, no individual study disproportionately influenced the pooled estimates, supporting the robustness and stability of the findings across all outcomes (Supplementary File S1).

## 4. Discussion

In this systematic review and meta-analysis, the germline *RET* M918T pathogenic variant was associated with significantly higher pooled unadjusted odds of lymph node metastasis, metastatic disease, advanced-stage disease, and mortality in the overall analysis among patients with hMTC. Disease-specific mortality was significantly higher among M918T carriers, whereas the association with all-cause mortality was not statistically significant; the between-subgroup difference was also not significant. Comparator groups comprised other germline *RET* pathogenic variants but varied across studies, including individual variants, codon-based groups, and genotype-based risk categories [12,13,14,15,16,17,18,19,20], as detailed in Supplementary Table S2. Consequently, the pooled estimates represent comparisons across heterogeneous study-specific comparator groups rather than with a single uniform reference variant. Although the magnitude varied across outcomes, all pooled point estimates indicated poorer outcomes among M918T carriers. These findings support the prognostic relevance of M918T but should be interpreted as unadjusted prognostic associations rather than evidence of an independent causal effect.

The biological plausibility of these associations is supported by the distinct molecular effects of M918T. The *RET* proto-oncogene encodes the *RET* receptor, a transmembrane receptor tyrosine kinase involved in cellular proliferation, differentiation, migration, and survival [4,29]. Under physiological conditions, the binding of a GDNF family ligand to its cognate GFRα co-receptor promotes *RET* dimerization and trans-autophosphorylation, thereby initiating downstream signaling [29]. Activating germline *RET* pathogenic variants produce constitutive signaling through domain-specific mechanisms [29]. Variants affecting extracellular cysteine residues, particularly codon 634, promote aberrant intermolecular disulfide-bond formation and ligand-independent *RET* dimerization [29,30]. In contrast, M918T lies within the intracellular tyrosine kinase domain and selectively enhances autophosphorylation at Tyr1062 relative to MEN2A-associated *RET* mutants [31]. It also promotes constitutive, dimerization-independent kinase activation [29,30,32] and altered substrate specificity [33]. Enhanced RAS–MAPK and PI3K–AKT signaling may subsequently promote cell proliferation, survival, and motility [29,31,32]. These mechanisms provide a biological rationale for the unfavorable phenotype observed among M918T carriers.

The pooled findings are broadly consistent with previous evidence linking germline M918T to early disease onset and a severe clinical phenotype. M918T is the predominant germline *RET* pathogenic variant associated with MEN3, which is characterized by early-onset MTC and a substantial risk of metastatic disease [2,3,4,34]. Long-term genotype–phenotype studies have shown that the underlying germline *RET* pathogenic variant influences age-related disease penetrance and age at onset, with M918T associated with the earliest clinical presentation [6,7,8,34]. Several included cohorts also reported higher frequencies of lymph node involvement, metastatic disease, advanced-stage disease, or mortality among M918T carriers than among their comparator groups [12,13,14,15,16,17,18,19,20]. Nevertheless, because M918T is closely associated with the broader MEN3 phenotype, its prognostic contribution cannot be fully separated from syndrome-related characteristics and differences in diagnostic pathways.

The present findings should also be considered alongside the large longitudinal cohort study by Machens et al., which suggested that germline *RET* pathogenic variants primarily determine tumor onset and age-related penetrance rather than intrinsic tumor aggressiveness [20]. The two studies addressed different questions: Machens et al. used time-to-event analyses to distinguish tumor onset and penetrance from invasion and metastasis across *RET* risk categories, whereas this meta-analysis compared the occurrence of adverse outcomes using aggregate event counts. The pooled ORs did not account for age at disease onset, duration of tumor growth, or time to outcome. Differences in age at diagnosis, case ascertainment, thyroidectomy timing and indication, follow-up duration, treatment era, and comparator composition may therefore have contributed to the observed associations. M918T may consequently provide prognostic information but cannot be regarded as an independently established determinant of intrinsic tumor aggressiveness.

These observations are consistent with the American Thyroid Association genotype-based risk classification, which places M918T in the highest-risk category (ATA-HST) and recommends thyroidectomy as soon as possible during the first year of life, potentially within the first months [1]. Studies of young carriers of germline *RET* pathogenic variants have demonstrated favorable outcomes when thyroidectomy is performed before clinically apparent or node-positive disease, although most evidence derives from MEN2A or other non-M918T variants [10,35,36]. These studies support early genotype-informed intervention but do not directly quantify the effect of prophylactic thyroidectomy among M918T carriers. Moreover, MTC may already be present at a very young age in some carriers; thus, surgery performed early in life may not always be prophylactic in a strictly pathological sense. Differences in thyroidectomy timing and indication may therefore have influenced outcomes across variant groups.

The review focused on MTC-related outcomes rather than the full clinical spectrum of MEN3. Pheochromocytoma, mucosal neuromas, gastrointestinal ganglioneuromatosis, marfanoid habitus, and other manifestations may influence diagnostic pathways, surveillance, overall health, and mortality independently of MTC severity [2,3,7,11]. Their effects could not be separated because they were not consistently reported by variant group or included in adjusted analyses, a limitation particularly relevant to all-cause mortality. Considerable phenotypic heterogeneity also exists among M918T carriers. Genetic modifiers, epigenetic mechanisms, and environmental exposures may contribute to biological variability [29,34], while age and stage at diagnosis, case ascertainment, thyroidectomy timing, biochemical and imaging findings, and access to early intervention may further affect observed prognosis. Germline *RET* variant status should therefore complement, rather than replace, established clinicopathological factors. Although molecular characterization is increasingly relevant to advanced MTC harboring *RET* alterations in the era of selective *RET* inhibition [2,37,38], the included studies did not evaluate genotype-directed surveillance or treatment strategies. These findings should not, by themselves, modify established recommendations for surgery, surveillance, or systemic treatment.

Key strengths include the quantitative synthesis of a rare hereditary cancer across international cohorts, adherence to a prospectively registered protocol and PRISMA 2020, and formal assessment with the Quality in Prognosis Studies tool. Direct comparisons between M918T carriers and study-specific comparator groups, together with transparent documentation of comparator definitions, thyroidectomy indications, case ascertainment, and potentially overlapping populations, improved interpretability. Harmonized outcome definitions and sensitivity analyses further supported the robustness of the principal findings.

Several limitations should be considered. First, all included studies were observational cohorts, and the pooled ORs were calculated from unadjusted aggregate event counts rather than adjusted individual-level estimates. Residual confounding by age, disease stage at diagnosis, follow-up duration, treatment era, thyroidectomy timing, and other clinical factors cannot be excluded. The ORs also did not account for time to outcome and should not be interpreted as hazard ratios, incidence-rate estimates, or evidence of a temporal disease-progression sequence. An independent causal effect of M918T could therefore not be established.

Second, comparator groups, outcome definitions, and assessment time points varied across studies. The non-M918T groups included different individual germline *RET* pathogenic variants, codon-based groups, and genotype-based risk categories, while substantial heterogeneity in the lymph node metastasis analysis indicated additional clinical or methodological variability. Metastatic disease combined M1 disease at diagnosis, distant metastasis during follow-up, and distant recurrence; advanced-stage disease may have been classified using different American Joint Committee on Cancer editions; and mortality comprised disease-specific and all-cause definitions. Although outcomes were harmonized before synthesis and mortality was examined by definition, residual heterogeneity remained. The small number of studies precluded reliable subgroup analyses or meta-regression by individual comparator variant or risk category.

Third, case ascertainment and thyroidectomy timing and indication were not consistently reported separately by variant group. Clinically apparent index cases may have had more advanced disease at diagnosis, whereas family-screened carriers may have been identified earlier and undergone thyroidectomy before clinically evident disease. This concern is particularly relevant to MEN3 because many M918T cases arise de novo and cannot be identified through conventional cascade testing before the recognition of the clinical phenotype [3,7,11]. Stratified analyses by index status or therapeutic versus prophylactic thyroidectomy were not feasible; consequently, the pooled estimates may partly reflect differences in disease detection and opportunities for early intervention rather than genotype alone.

Finally, some analyses included few studies and events, limiting precision and generalizability and preventing formal assessment of publication bias because fewer than 10 studies contributed to each outcome. Although potentially overlapping populations were evaluated using study setting, recruitment period, authorship, participant characteristics, *RET* variant distribution, and outcomes, residual overlap could not be excluded without individual identifiers. Eligibility was restricted to English-language full-text articles published from January 2000 onward; despite the absence of language or year filters during database searching, these criteria may have introduced language or selection bias. Inconsistent reporting of MEN3-associated manifestations also limited separation of MTC-related and syndrome-related contributions, particularly to all-cause mortality. The pooled estimates should therefore be interpreted as prognostic associations relative to heterogeneous study-specific comparator groups rather than as evidence that M918T independently causes adverse outcomes.

## 5. Conclusions

This systematic review and meta-analysis found that the germline *RET* M918T pathogenic variant was associated with significantly higher unadjusted odds of lymph node metastasis, metastatic disease, advanced-stage disease, and mortality in patients with hMTC. These findings support the prognostic relevance of M918T and reinforce the clinical importance of genotype–phenotype correlations. However, the evidence was derived from a limited number of observational cohort studies, predominantly retrospective in design, and was subject to clinical heterogeneity and potential residual confounding by age, case ascertainment, surgical timing, and thyroidectomy indication. The findings should therefore be interpreted as prognostic associations rather than evidence of an independent causal effect or a basis for modifying current guideline-based management. Further multicenter prospective cohort or registry-based studies using standardized outcome definitions, detailed genotype and treatment data, and adequate longitudinal follow-up are needed to clarify the independent prognostic contribution of M918T.

## Figures and Tables

**Table 1:** Characteristics of studies included in the systematic review and meta-analysis.

Study	Country	Study Design	Study Period	Total Cohort Size (*n*) ^a^	Study Population Characteristics	Age at MTC Diagnosis, Years ^b^	Age at Thyroidectomy, Years ^b^	Genetic Testing Method	Specimen Source	*RET* M918T Variant, *n*	Other Germline *RET* Variants, *n*	Follow-Up, Years	Outcomes Reported
Yip et al., 2003 [12]	USA	Retrospective cohort	1951–2002	86	Mixed pediatric/adult; MEN2A, MEN2B, and FMTC	NR	M918T: median 13.5 (5.0–25.5); other *RET* variants: Level 1, 41.5 (17.4–78.2), and Level 2, 22.9 (5.2–60.4)	DNA sequencing and allele-specific hybridization	Peripheral blood	10	68	NR	LNM; metastatic disease ^c^; advanced-stage disease ^d^; mortality ^e^
Rohmer et al., 2011 [13]	France	Multicenter retrospective cohort	1977–2006	170	Pediatric/adolescent/young adult (<21 years); hMTC	NR	M918T: mean 7.5 ± 5.1; other *RET* variants: Class A, 10.7 ± 4.5; Class B, 9.6 ± 4.1; Class C, 8.4 ± 4.0	PCR and direct DNA sequencing	Peripheral blood	24	146	Median 5.8 (0.01–28.7)	LNM
Jung et al., 2018 [14]	South Korea	Retrospective cohort	1982–2012	57	Mixed pediatric/adult; MEN2A, MEN2B, and FMTC	M918T: mean 19.7 ± 1.5; other *RET* variants: moderate risk, 42.0 ± 12.4; high risk, 33.6 ± 11.5	NR	Direct DNA sequencing	NR	3	42	Median 6.75	LNM; metastatic disease ^c^; mortality ^e^
Spanheimer et al., 2019 [15]	USA	Retrospective single-center cohort	1986–2017	66	Mixed pediatric/adult; familial MTC	NR	Overall: median 35.2 (3.4–72.3); M918T and other *RET* variants: NR separately	NR	NR	8	58	Median 9.3 (0.3–31.5)	Metastatic disease ^c^
Raue et al., 2019 [16]	Germany	Retrospective cohort	1979–2017	263	Mixed pediatric/adult; MEN2	NR	M918T: mean 14.9 ± 9.3; other *RET* variants: moderate risk, 35.3 ± 18.8; high risk, 23.0 ± 15.7	NR	NR	21	242	Mean 12.9 ± 9.8	Metastatic disease ^c^; advanced-stage disease ^d^; mortality ^e^
Kuhlen et al., 2020 [17]	Germany	Prospective multicenter registry cohort	1997–2019	57	Pediatric/adolescent (0–18 years); MEN2A and MEN2B	M918T: mean 9.2 ± 5.3; other *RET* variants: mean 9.6 ± 4.7	NR	NR	NR	27	27	Median 5 (0–19)	LNM; metastatic disease ^c^; mortality ^e^
Valiveru et al., 2021 [18]	India	Retrospective cohort	2004–2018	55	Mixed pediatric/adult; MEN2A, MEN2B, and FMTC	M918T: median 15; other *RET* variants: high risk, 25 (IQR 16–40); moderate risk, 24 (IQR 22–34)	NR	NR	Peripheral blood	2	53	Median 4 (1–16)	Advanced-stage disease ^d^
Di Benedetto et al., 2025 [19]	Italy	Retrospective cohort	1980–2018	23	Pediatric/adolescent (≤19 years); MEN2 carriers	NR	M918T: 12; other *RET* variants: high risk, median 16 (5–19); moderate risk, median 14 (4–19)	PCR and direct DNA sequencing	Peripheral blood	1	22	Median 9.7 (2–36)	LNM; metastatic disease ^c^; advanced-stage disease ^d^; mortality ^e^
Machens et al., 2026 [20]	Germany	Retrospective cohort	1985–2025	708	Mixed pediatric/adult; germline RET variant carriers with MEN2	NR	M918T: median 12 (95% CI 3–17); other *RET* variants: high risk, 16 (6–31); intermediate risk, 28 (10–43); low risk, 38 (21.8–55)	NR	NR	55	653	NR	LNM; metastatic disease ^c^

Abbreviations: AJCC, American Joint Committee on Cancer; CI, confidence interval; FMTC, familial medullary thyroid carcinoma; hMTC, hereditary medullary thyroid carcinoma; IQR, interquartile range; LNM, lymph node metastasis; MEN2, multiple endocrine neoplasia type 2; MTC, medullary thyroid carcinoma; NR, not reported; PCR, polymerase chain reaction. The study-specific comparator variants and risk categories are detailed in Supplementary Table S2. ^a^ Total cohort size represents the eligible hMTC or germline *RET*-positive cohort. The M918T and other germline *RET* pathogenic variant groups represent the variant-specific samples included in the meta-analysis and may not sum to the total cohort size. Outcome-specific denominators may vary because of missing or unavailable data. ^b^ None of the included studies explicitly reported age at germline *RET* variant identification; the values presented refer specifically to age at MTC diagnosis or age at thyroidectomy, as indicated. ^c^ Metastatic disease was defined as M1 disease at diagnosis, distant metastasis during follow-up, or distant recurrence. ^d^ Advanced-stage disease was defined as AJCC stage III–IV. ^e^ Mortality was classified as disease-specific or all-cause mortality.

## Data Availability

The data supporting the findings of this systematic review and meta-analysis are available within the article and its Supplementary Materials. The extracted data used for the meta-analysis are available from the corresponding author upon reasonable request.

## Supplementary Materials

The following supporting information can be downloaded at https://www.mdpi.com/article/10.3390/medicina62091636/s1. Supplementary Table S1: Search strategies used for each database; Supplementary Table S2: Study-specific other germline *RET* variant comparators, thyroidectomy indications, and case ascertainment; Supplementary Table S3: Risk-of-bias assessment of the included studies using the Quality in Prognosis Studies (QUIPS) tool; Supplementary File S1: Leave-one-out sensitivity analyses; Supplementary File S2: PRISMA 2020 Checklist [21]; Supplementary File S3: PRISMA 2020 for Abstracts Checklist.
