# Supplementary material for: Clinical Outcomes of the Germline RET M918T Pathogenic Variant in Hereditary Medullary Thyroid Carcinoma: A Systematic Review and Meta-Analysis
Source: Medicina (Kaunas). 2026 Aug 26;62(9):1636. doi: 10.3390/medicina62091636 (PMC13609479; doi:10.3390/medicina62091636)
Supplement: Supplementary file 1 [file medicina-62-01636-s001.zip › Supplementary_File_S1_Leave-One-Out_Sensitivity_Analysis.pdf]

Supplementary File S1. Leave-one-out sensitivity analyses

1. Lymph node metastasis

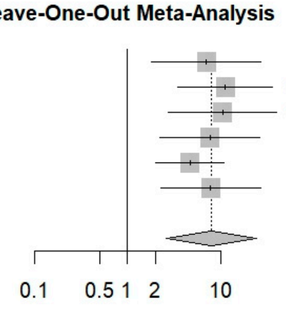

Study	OR	95% CI	P-value	Tau2	Tau	I2
Omitting Kuhlen et al., 2020	7.01	[1.81; 27.06]	0.0047	1.5033	1.2261	71.7%
Omitting Yip et al., 2003	11.14	[3.48; 35.72]	< 0.0001	0.9828	0.9913	69.4%
Omitting Machens et al.,2026	10.39	[2.72; 39.69]	0.0006	1.3446	1.1596	64.8%
Omitting Jung et al.,2018	7.63	[2.21; 26.33]	0.0013	1.3848	1.1768	74.9%
Omitting Rohmer et al., 2011	4.65	[1.98; 10.88]	0.0004	0.3448	0.5872	28.8%
Omitting Di Benedetto et al., 2025	7.80	[2.29; 26.55]	0.0010	1.3679	1.1696	75.1%
**Random effects model**	**7.86**	**[2.58; 23.94]**	**0.0003**	**1.1822**	**1.0873**	**69.1%**

2. Metastatic disease

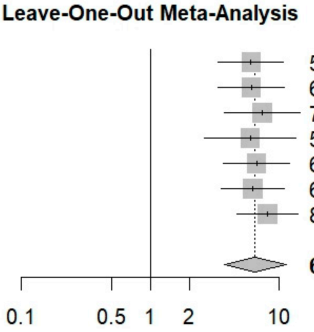

Study	OR	95% CI	P-value	Tau2	Tau	I2
Omitting Spanheimer et al., 2019	5.99	[3.36; 10.70]	< 0.0001	0.0748	0.2734	0.9%
Omitting Kuhlen et al., 2020	6.06	[3.36; 10.92]	< 0.0001	0.0838	0.2895	7.5%
Omitting Yip et al., 2003	7.34	[3.75; 14.35]	< 0.0001	0.1407	0.3750	8.0%
Omitting Machens et al., 2026	5.93	[2.60; 13.51]	< 0.0001	0.1928	0.4391	9.6%
Omitting Jung et al., 2018	6.63	[3.69; 11.90]	< 0.0001	0.0920	0.3033	19.1%
Omitting Di Benedetto et al., 2025	6.21	[3.52; 10.95]	< 0.0001	0.0741	0.2722	4.4%
Omitting Raue et al., 2019	8.09	[4.69; 13.96]	< 0.0001	0	0	0%
**Random effects model**	**6.50**	**[3.72; 11.37]**	**< 0.0001**	**0.0723**	**0.2688**	**4.8%**

3. Advanced-stage disease

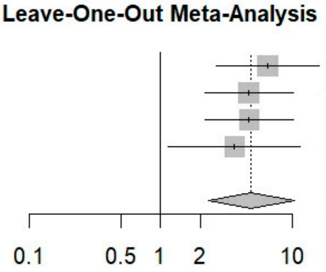

Study	OR	95% CI	P-value	Tau2	Tau	I2
Omitting Yip et al., 2003	6.50	[2.63; 16.06]	< 0.0001	0	0	0%
Omitting Valiveru et al., 2021	4.67	[2.15; 10.14]	< 0.0001	0	0	0%
Omitting Di Benedetto et al., 2025	4.70	[2.18; 10.17]	< 0.0001	0	0	0%
Omitting Raue et al., 2019	3.63	[1.14; 11.57]	0.0291	0	0	0%
**Random effects model**	**4.88**	**[2.30; 10.33]**	**< 0.0001**	**0**	**0**	**0%**

4. Mortality

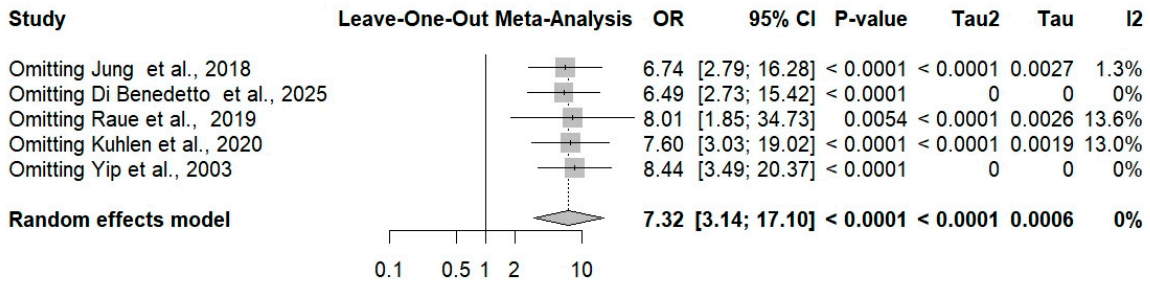

Study
Leave-One-Out Meta-Analysis
OR
95% CI
P-value
Tau2
Tau
I2
Omitting Jung et al., 2018 6.74 [2.79; 16.28] < 0.0001 < 0.0001 0.0027 1.3%
Omitting Di Benedetto et al., 2025 6.49 [2.73; 15.42] < 0.0001 0 0 0%
Omitting Raue et al., 2019 8.01 [1.85; 34.73] 0.0054 < 0.0001 0.0026 13.6%
Omitting Kuhlen et al., 2020 7.60 [3.03; 19.02] < 0.0001 < 0.0001 0.0019 13.0%
Omitting Yip et al., 2003 8.44 [3.49; 20.37] < 0.0001 0 0 0%
Random effects model 7.32 [3.14; 17.10] < 0.0001 < 0.0001 0.0006 0%
0.1
0.5
1
2
10
