# Supplementary material for: Clinical Outcomes of the Germline RET M918T Pathogenic Variant in Hereditary Medullary Thyroid Carcinoma: A Systematic Review and Meta-Analysis
Source: Medicina (Kaunas). 2026 Aug 26;62(9):1636. doi: 10.3390/medicina62091636 (PMC13609479; doi:10.3390/medicina62091636)
Supplement: Supplementary file 1 [file medicina-62-01636-s001.zip › Supplementary_Table_S1_Search_Strategy.pdf]

Supplementary Table S1. Search strategies used for each database

Database	Search strategy	Records (n)
PubMed	("Thyroid Neoplasms"[Mesh] OR "Carcinoma, Medullary"[Mesh] OR "medullary thyroid carcinoma"[Title/Abstract] OR "medullary thyroid cancer"[Title/Abstract] OR MTC[Title/Abstract]) AND ("Proto-Oncogene Proteins c-ret"[Mesh] OR RET[Title/Abstract] OR "RET mutation"[Title/Abstract] OR "RET proto-oncogene"[Title/Abstract]) AND ("Prognosis"[Mesh] OR "Survival Analysis"[Mesh] OR "Disease-Free Survival"[Mesh] OR "Treatment Outcome"[Mesh] OR prognosis[Title/Abstract] OR survival[Title/Abstract] OR outcome[Title/Abstract] OR recurrence[Title/Abstract] OR metastasis[Title/Abstract] OR mortality[Title/Abstract]) NOT (review[Publication Type])	871
Scopus	TITLE-ABS-KEY ("medullary thyroid carcinoma" OR "medullary thyroid cancer" OR MTC) AND TITLE-ABS-KEY ("RET mutation" OR "RET proto-oncogene" OR "germline RET" OR "somatic RET") AND TITLE-ABS-KEY (prognosis OR survival<br><br>OR "disease-free survival" OR "overall survival" OR recurrence OR metastasis OR mortality) AND NOT TITLE-ABS-KEY (review OR "case report")	359
Embase	('medullary thyroid carcinoma'/exp OR 'medullary thyroid carcinoma':ti,ab,kw OR 'medullary thyroid cancer':ti,ab,kw) AND ('ret proto oncogene'/exp OR 'RET mutation':ti,ab,kw OR 'germline RET':ti,ab,kw OR 'somatic RET':ti,ab,kw) AND ('prognosis'/exp OR 'overall survival'/exp OR 'disease free survival'/exp OR prognosis:ti,ab,kw OR survival:ti,ab,kw OR recurrence:ti,ab,kw OR metastasis:ti,ab,kw) NOT ('review'/it OR 'case report'/it OR 'conference abstract'/it)	174

Footnotes: Searches were conducted from database inception to 30 May 2026.

Abbreviations: MTC, medullary thyroid carcinoma; RET, rearranged during transfection proto-oncogene.
