# Supplementary material for: Clinical Outcomes of the Germline RET M918T Pathogenic Variant in Hereditary Medullary Thyroid Carcinoma: A Systematic Review and Meta-Analysis
Source: Medicina (Kaunas). 2026 Aug 26;62(9):1636. doi: 10.3390/medicina62091636 (PMC13609479; doi:10.3390/medicina62091636)
Supplement: Supplementary file 1 [file medicina-62-01636-s001.zip › Supplementary_Table_S2_RET_Comparator_Thyroidectomy_Ascertainment.pdf]

Supplementary Table S2. Study-Specific Other Germline *RET* Variant Comparators, Thyroidectomy Indications, and Case Ascertainment

Study	Other germline *RET* variants/risk categories	Prophylactic thyroidectomy, n	Therapeutic thyroidectomy, n	Index cases, n	Family-screened/non-index cases, n	Notes
Yip et al., 2003	Levels 1–2: codons 609, 611, 618, 620, 634, 804, and 891	15[a]	10[a]	NR	NR	Surgical indication was reported only for the pediatric subgroup.
Rohmer et al., 2011	ATA Classes A–C: codons 531, 611, 618, 620, 634, 666–667, 768, 790, 804, 844, and 891	NR	NR	NR	NR	Included *RET* carriers aged <21 years who underwent thyroidectomy; indication and ascertainment were not quantified.
Jung et al., 2018	ATA moderate-risk variants and high-risk C634	NR	NR	46	11	Index cases were diagnosed through clinically apparent tumors; screened cases were identified through familial screening.
Spanheimer et al., 2019	All other familial MTC patients, including specified non-M918T variants and clinically familial cases without a specified *RET* variant	14	52[b]	NR	NR	All prophylactic-intent patients had MTC on postoperative histology.
Raue et al., 2019	ATA high-risk C634/A883F and moderate-risk *RET* variants	NR	NR	100	163	Surgical pathways were incompletely reported for the entire cohort; index status was reported separately.
Kuhlen et al., 2020	MEN2A-associated variants, predominantly C634; additional variants included codons 790 and 791; variant data were unavailable for some patients	25	21	NR	NR	Surgical indication was unavailable or unclassified for 11 patients.
Valiveru et al., 2021	High-risk C634R/Y/G/S/T and moderate-risk D631Y/C611Y/V804M	9	35[c]	23	32	Forty-four of 55 patients underwent thyroidectomy.
Di Benedetto et al., 2025	High-risk C634 and moderate-risk C618/C620	NR	NR	2	21	All 23 patients underwent thyroidectomy; indication was not explicitly classified.
Machens et al., 2026	High-, intermediate-, and low-category non-M918T *RET* variants	NR	NR	222	486	Prophylactic versus therapeutic indication was not reported.

[a] Reported only for the pediatric subgroup (n = 25), not the entire cohort.

[b] Refers to patients with known MTC before thyroidectomy rather than an explicitly labeled therapeutic thyroidectomy group.

[c] Calculated as the number of operated patients minus those undergoing truly prophylactic thyroidectomy (44 − 9 = 35).

Comparator groups were defined according to the original studies. Prophylactic/therapeutic thyroidectomy status and index/family-screened status were extracted independently and were not considered interchangeable. A family-screened carrier could undergo therapeutic thyroidectomy if MTC was clinically, biochemically, or radiologically apparent before surgery.

Abbreviations: ATA, American Thyroid Association; MEN2, multiple endocrine neoplasia type 2; MTC, medullary thyroid carcinoma; NR, not reported; *RET*, rearranged during transfection.
