# Supplementary material for: Clinical Outcomes of the Germline RET M918T Pathogenic Variant in Hereditary Medullary Thyroid Carcinoma: A Systematic Review and Meta-Analysis
Source: Medicina (Kaunas). 2026 Aug 26;62(9):1636. doi: 10.3390/medicina62091636 (PMC13609479; doi:10.3390/medicina62091636)
Supplement: Supplementary file 1 [file medicina-62-01636-s001.zip › Supplementary_Table_S3_Risk_of_Bias_Assessment.pdf]

Supplementary Table S3. Risk-of-bias assessment of the included studies using the Quality in Prognosis Studies (QUIPS) tool.

Study	Study participation	Study attrition	Prognostic factor measurement	Outcome measurement	Study confounding	Statistical analysis and reporting	Overall risk of bias
Yip et al., 2003 [12]	Moderate	Moderate	Low	Low	Moderate	Moderate	Moderate
Rohmer et al., 2011 [13]	Low	Low	Low	Low	Low	Low	Low
Jung et al., 2018 [14]	Moderate	Moderate	Low	Low	Moderate	Moderate	Moderate
Spanheimer et al., 2019 [15]	Low	Low	Low	Low	Low	Low	Low
Raue et al., 2019 [16]	Low	Moderate	Low	Low	Low	Low	Low
Kuhlen et al., 2020 [17]	Low	Moderate	Low	Low	Moderate	Low	Moderate
Valiveru et al., 2021 [18]	Moderate	Moderate	Low	Low	High	Moderate	Moderate
Di Benedetto et al., 2025 [19]	Moderate	Moderate	Low	Low	High	Moderate	Moderate
Machens et al., 2026 [20]	Low	Moderate	Low	Low	Moderate	Moderate	Moderate

Abbreviations: QUIPS, Quality in Prognosis Studies.

Risk-of-bias ratings were assigned according to the QUIPS domains and categorized as low, moderate, or high risk of bias.

Overall QUIPS risk-of-bias summary

Overall risk of bias	Number of studies
Low	3
Moderate	6
High	0
